# Sonodynamic therapy in combination with photodynamic therapy shows enhanced long-term cure of brain tumor

**DOI:** 10.1038/s41598-020-78153-0

**Published:** 2020-12-11

**Authors:** Ballav M. Borah, Joseph Cacaccio, Farukh A. Durrani, Wiam Bshara, Steven G. Turowski, Joseph A. Spernyak, Ravindra K. Pandey

**Affiliations:** 1 Photolitec, LLC, 73 High Street, Buffalo, NY 14203 USA; 2 Department of Cell Stress Biology, Photodynamic Therapy Center, Roswell Park Comprehensive Cancer Center, Buffalo, NY 14263 USA; 3 Department of Pathology, Pathology Network Shared Resource, Roswell Park Comprehensive Cancer Center, Buffalo, NY 14263 USA; 4 Translational Imaging Shared Resource, Cell Stress Biology, Roswell Park Comprehensive Cancer Center, Buffalo, NY 14263 USA

**Keywords:** Cancer, Nanoscience and technology

## Abstract

This article presents the construction of a multimodality platform that can be used for efficient destruction of brain tumor by a combination of photodynamic and sonodynamic therapy. For in vivo studies, U87 patient-derived xenograft tumors were implanted subcutaneously in SCID mice. For the first time, it has been shown that the cell-death mechanism by both treatment modalities follows two different pathways. For example, exposing the U87 cells after 24 h incubation with HPPH [3-(1′-hexyloxy)ethyl-3-devinyl-pyropheophorbide-a) by ultrasound participate in an electron-transfer process with the surrounding biological substrates to form radicals and radical ions (Type I reaction); whereas in photodynamic therapy, the tumor destruction is mainly caused by highly reactive singlet oxygen (Type II reaction). The combination of photodynamic therapy and sonodynamic therapy both in vitro and in vivo have shown an improved cell kill/tumor response, that could be attributed to an additive and/or synergetic effect(s). Our results also indicate that the delivery of the HPPH to tumors can further be enhanced by using cationic polyacrylamide nanoparticles as a delivery vehicle. Exposing the nano-formulation with ultrasound also triggered the release of photosensitizer. The combination of photodynamic therapy and sonodynamic therapy strongly affects tumor vasculature as determined by dynamic contrast enhanced imaging using HSA-Gd(III)DTPA.

## Introduction

Glioblastoma multiforme (GBM) is a highly aggressive and deeply penetrating brain cancer that is notoriously difficult to treat^1–4^. The standard treatment for glioblastoma is to remove the tumor mass, clean the margins with radiation therapy, and then administrator either the alkylating agent Temozolomide (TMZ) or Bevacizumab, an inhibitor of vascular endothelial growth factor (VEGF)]^5–7^. These mainstays of modern oncology have distinct limitations. While surgical resection of the mass can debulk the tumor, GBM has notoriously poorly defined margins. Therefore, it is imperative that any surgical option is followed up by cleaning the margins. The most common way to do this is with radiation therapy, which, while effective, has toxicity issues and limited efficacy on recurrent tumors^8,9^. Recent cancer statistics indicate that GBM has a near universal recurrence rate and a 5-year survival rate of < 10%^10,11^. Finally, chemotherapy damages the immune system and produces serious side effects by killing healthy cells^12–15^. There is a clear and present need for significant improvement in treatments for both newly diagnosed and recurrent GBM. Notably, there have been multiple clinical studies investigating photonics-based PDT and fluorescence guided resection (FGR) to better clean the margins and improve survival^16–26^. For these reasons, 5-ALA (a pro-drug) for fluorescence-guided PDT in grade III and IV gliomas has been approved by FDA^18^. Additionally, these early trials have demonstrated that PDT is safe and potentially very effective for the treatment of GBM. One interesting clinical trial in Australia demonstrated that Photodynamic therapy improved prognosis in newly diagnosed GBM^22^. An undergoing clinical trial in Japan which utilized talaporfin sodium resulted in an improved mean overall survival of 24.8 months in newly diagnosed GBM^26,27^. However, one major drawback of PDT is the limited depth of penetration of the light. Deeply seeded or highly invasive tumors will not respond well to PDT. Therefore, exposing the tumor to light in combination with ultrasound, which provides deeper tissue penetration ability, should help destroy infiltrated GBM tumor cells, resulting to enhanced long term survival of cancer patients^28,29^.

SDT is a novel therapy that shares many of the advantages of PDT while improving on some of PDT’s drawbacks^30–33^. Both treatment modalities can be focused on a small region to prevent normal cell toxicity, or be used to destroy tumor resection margins and deeply seated tumors^34,35^. Mechanistically, PDT causes cascading biological responses to clear tumors, originating from the photosensitizer’s production of reactive oxygen species (ROS). Current mechanistic studies suggests that during SDT sonosensitizer produces ROS when in close proximity to a collapsing cavitation bubbles generated by ultrasonic wave^36^. The sensitizer‐derived ROS interact with a wide variety of targets, such as cytoskeletal proteins and cellular membranes, ultimately inducing cell death through apoptosis and necrosis^37–39^. Additionally, it has been shown that ROS generated from either PDT or SDT damages tumor vasculature networks leading to vasculature shutdown^40,41^. Where the two differ is that since SDT utilizes US instead of light, SDT is not hampered by a limited depth of penetration^30–33,42,43^. In a review of 2019, Lefond et al. showed that there have been several clinical studies that look into their combinational potential^44^. In fact, one study which investigated the combination of PDT and SDT to treat a wide variety of cancer diagnoses which have failed traditional therapies observed a significant improvement in survival^45^. Moreover, it has been shown that the porphyrin-based compounds used in PDT have the potential to induce a response in SDT as well^34,35^. Therefore by combining the two treatment modalities, using HPPH as an effective agent, the survival rate could be greatly improved.

We have previously shown that non-toxic, biodegradable PAA based NPs are suitable delivery vehicles for cancer-imaging and/or phototherapy agents to the tumor^46–49^. Additionally, we established that the NPs had a photo-triggered release mechanism of the payload when exposed to at least 2 J of light^30,46^. Furthermore, Huebsch et al*.* demonstrated that US waves interact with the NPs and can induce drug release while not damaging the particle or the drug^50^. Therefore, we were interested to investigate the benefit of HPPH, which is already in Phase II human clinical trials (HPPH has also received an orphan drug status for the treatment of esophageal cancer by FDA)^51^.

Herein, we demonstrate the use of HPPH as a viable sonosensitizer and photosensitizer to treat GBM. The rationale for combination therapy (PDT and SDT) is to use different modalities that provide complimentary mechanism(s) of localized cell death., thereby decreasing the likelihood of recurrent disease^,^^52,53^. In our present study, we have optimized the synthesis of PAA-based cationic NPs (PAA-NMe^3+^) for the localized delivery and US-triggered controlled release of HPPH, and demonstrate its benefit over Tween80 formulation for the treatment of brain cancer by a combination of PDT and SDT.

## Results and discussion

### Photosensitizer

3-(1′-Hexyloxy)ethyl-3-devinylpyropheophorbide-a (HPPH) was synthesized by following the methodology developed in our laboratory^54^.

### Amino functionalized cationic polyacrylamide (PAA-NMe_3_^+^) nanoparticles

**Figure 1:**
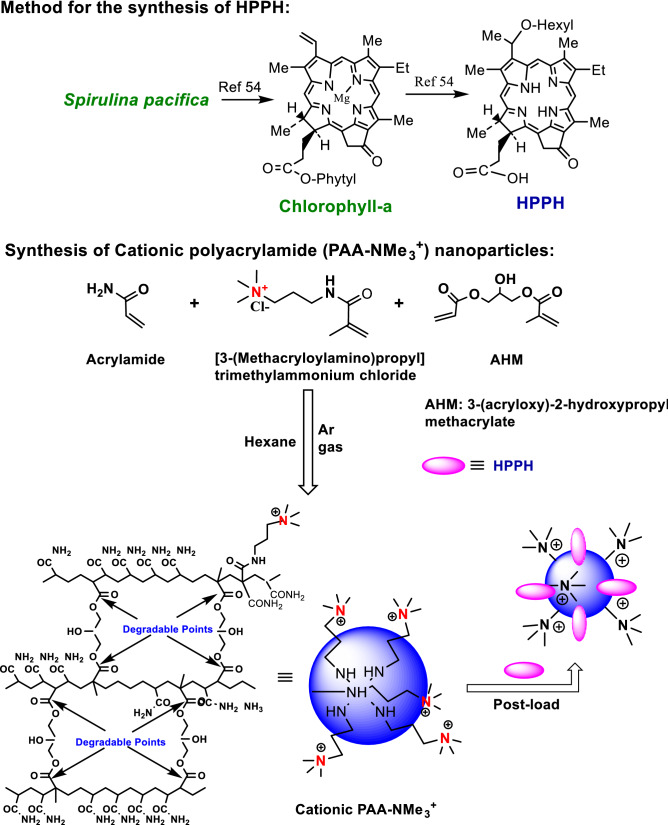
Synthesis of cationic polyacrylamide (PAA-NMe_3_^+^) nanoparticles post-loaded with HPPH.

Cationic PAA-NMe_3_^+^ nanoparticles with quaternary ammonium moieties were prepared following previously reported procedure^46^ as shown in Fig. 1.

### Design of ultra-sound (US) exposure setup

**Figure 2:**
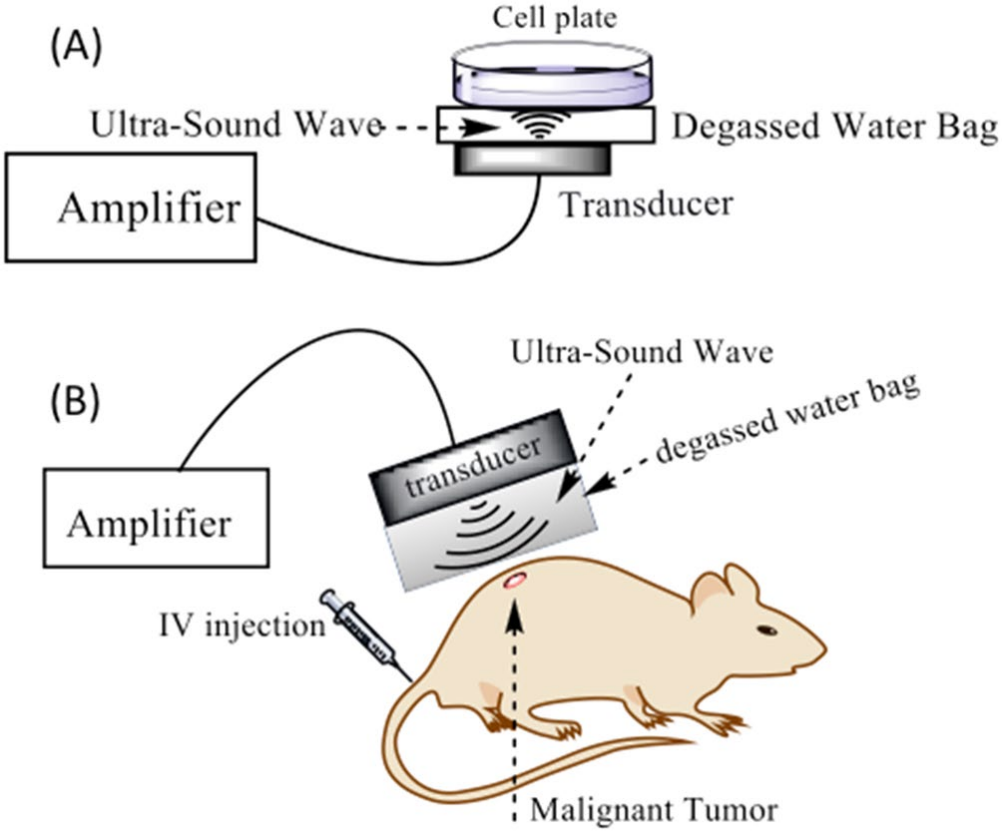
In-vitro and in-vivo experimental designs for sonodynamic therapy.

The 3.3 continuous focused ultra-sound (US) transducer is a circular single disk with diameter of 42.5 mm with a point target focus (PTF) of 76 mm. As shown in Fig. 2A, a degassed water bag is placed on top of the transducer (in-vitro SDT), to uniformly spread the US wave with minimal scattering and energy loss. For in-vivo SDT, the water bag is placed on top of the tumor (Fig. 2B). The idea behind this design is to have control over the increase in temperature, unlike commonly used US gels.

### Ultrasound (US) triggered release of sensitizers from the NPs

**Figure 3:**
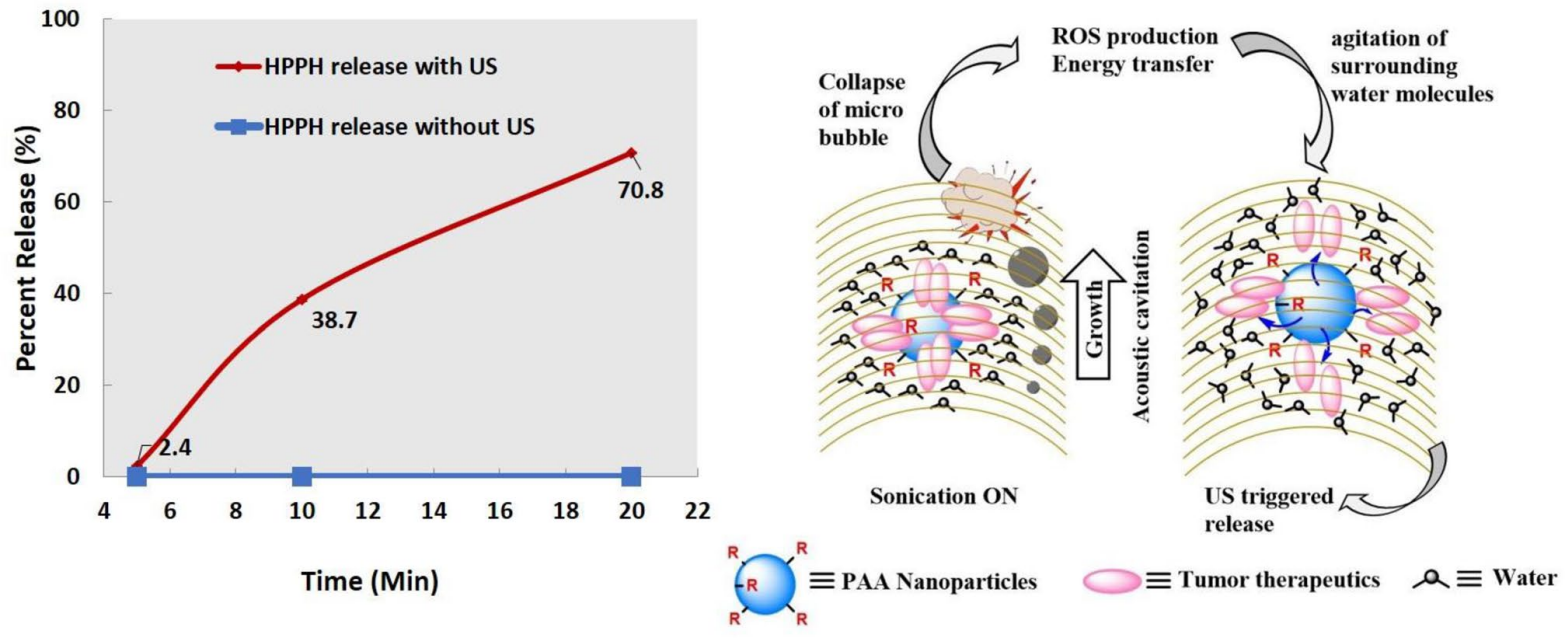
US-triggered release of HPPH post-loaded cationic PAA-NMe^3+^ nanoparticles was determined in 1% HSA-PBS. The HPPH (ε: 47,500 L/mol/cm at 661 nm) dispersed at a concentration of 20 μM in 1% HSA-PBS solution was irradiated with US at a dose of 0.5 W/cm^2^, 3.3 MHz at 5, 10 and 20 min. At each time point, the solution was filtered through a 100 KDa centrifuge filter. The concentration of HPPH in filtrates was determined spectrophotometrically, and the release of HPPH was plotted against time.

The cationic PAA-NMe^3+^ NPs post-loaded with HPPH were mixed with 1% (w/v) HSA in PBS. The absorbance spectrum of the solution was measured spectrophotometrically and was marked as the original absorbance. The PS (HPPH) in methanol and on post-loading to NPs did not show any significant difference in its extinction coefficient value and exhibited a small shift (3–4 nm) in its long wavelength absorption (See Fig. S1, Supporting Material). The US-triggered release was measured at 0.5 W/cm^2^, 3.3 MHz irradiation for 5 min, 10 min and 20 min. The temperature at the entire duration of irradiation was monitored by Omega Microprocessor Thermometer (*Model HH23, Type J-K-T Thermocouple*) and was found to be constant. The solutions after the US irradiation were then centrifuged in an Amicon Ultracel-4, 100 kDa centrifuge filter at 4000 RPM for 15 min. To measure the retained PS in the nanoparticles after two washes, the nanoparticles were reconstituted with 1% HSA in PBS, thoroughly mixed and the PS absorption was measured. The washes from both the steps were combined as the total filtrate. This signified the total PS released, and the absorption measurement of the retentate indicated the percentage of the PS that was retained. The results (Fig. 3) show that the US-triggered release can be achieved by irradiating the NPS for as low as 5 min at dose rate of 0.5 W/cm^2^, 3.3 MHz. The optimum release was achieved by US irradiation for 10 min at a dose rate of 0.5 W/cm^2^, 3.3 MHz. No release of PS was observed without US irradiation. On applying US, compression of the liquid is followed by expansion, in which a sudden pressure drops forms small, oscillating bubbles of gaseous substances. Due to this inertial cavitation; a process in which mechanical activation destroys the attractive forces of the molecule in liquid phase, the PS are being released from the hydrophobic pockets of cationic PAA-NMe_3_^+^ NPs as shown in the proposed US triggered release mechanism (Fig. 3). In an ongoing study, we have explored the utility of photo- and ultrasound triggered release of certain non-PDT related cancer-imaging and therapy agents (e.g., cyanine dyes, PET-imaging agents, curcumin, doxorubicin etc.) in tumors, and the initial results are promising. The optimized treatment parameters could also help in designing NPs to enhance the tumor-uptake of a variety of hydrophobic chemotherapy agents for enhanced long-term cure of cancer and metastasis with reduced toxicity.

### In vitro anticancer activity

To investigate the effect of ultrasound (US) in vitro efficacy, a cytotoxicity experiment was performed using either HPPH formulated in Tween or post-loaded in cationic NPs. The cells were harvested and counted utilizing trypan blue after a variety of exposure conditions. Cells that were exposed to ultrasound and HPPH (formulated either in Tween or NPs) had a significant decrease in viability compared to the control. HPPH formulated in NPs were significantly more efficacious than the Tween formulation at both SDT doses. Interestingly, there was no significant difference in cell viability between the two power levels in the HPPH NP group, suggesting that there could be a maximum exposure threshold limiting the efficacy, which needs to be explored.

**Figure 4:**
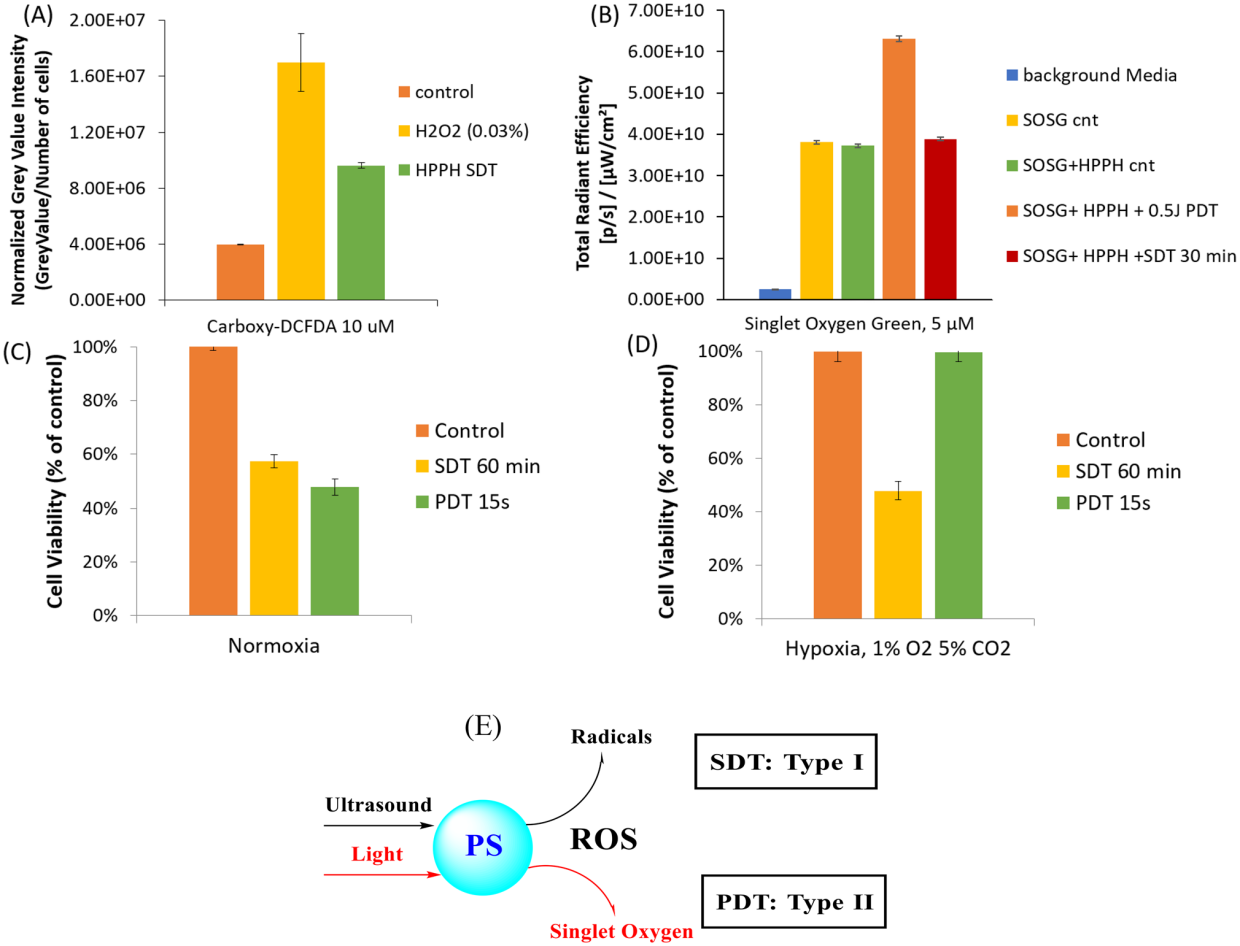
U87 cells incubated with 1 µM of HPPH formulated in either Tween or cationic PAA-NMe^3+^ and the number of cells was counted utilizing a trypan blue assay. To understand the type of ROS (singlet oxygen or radical ions) responsible of cell-kill mechanism the cells were incubated with carboxy-DCFDA and exposed to US or H_2_O_2_ (positive control for radical ions) (**A**) in vitro ROS measurement by Carboxy-DCFDA. Cells were imaged with fluorescent microscope and analyzed using ImageJ Grey Value intensity of the photosensitizer then normalized to number of cells. For detection of single oxygen, SOSG (a singlet oxygen quencher) was added to a test tube along with 10 µM of HPPH and exposed to light or US. The resulting fluorescence of SOSG under the different reactions was measured and graphed (**B**). To further confirm that SDT does not utilize singlet oxygen, the U87 cells were either grown in normoxia (**C**) or hypoxia environment (**D**) with 1 µM of HPPH. (**E**) Mechanisms for the formation of reactive oxygen species by exciting the PS with either light (Type I) or ultrasound (Type II).

**Figure 5:**
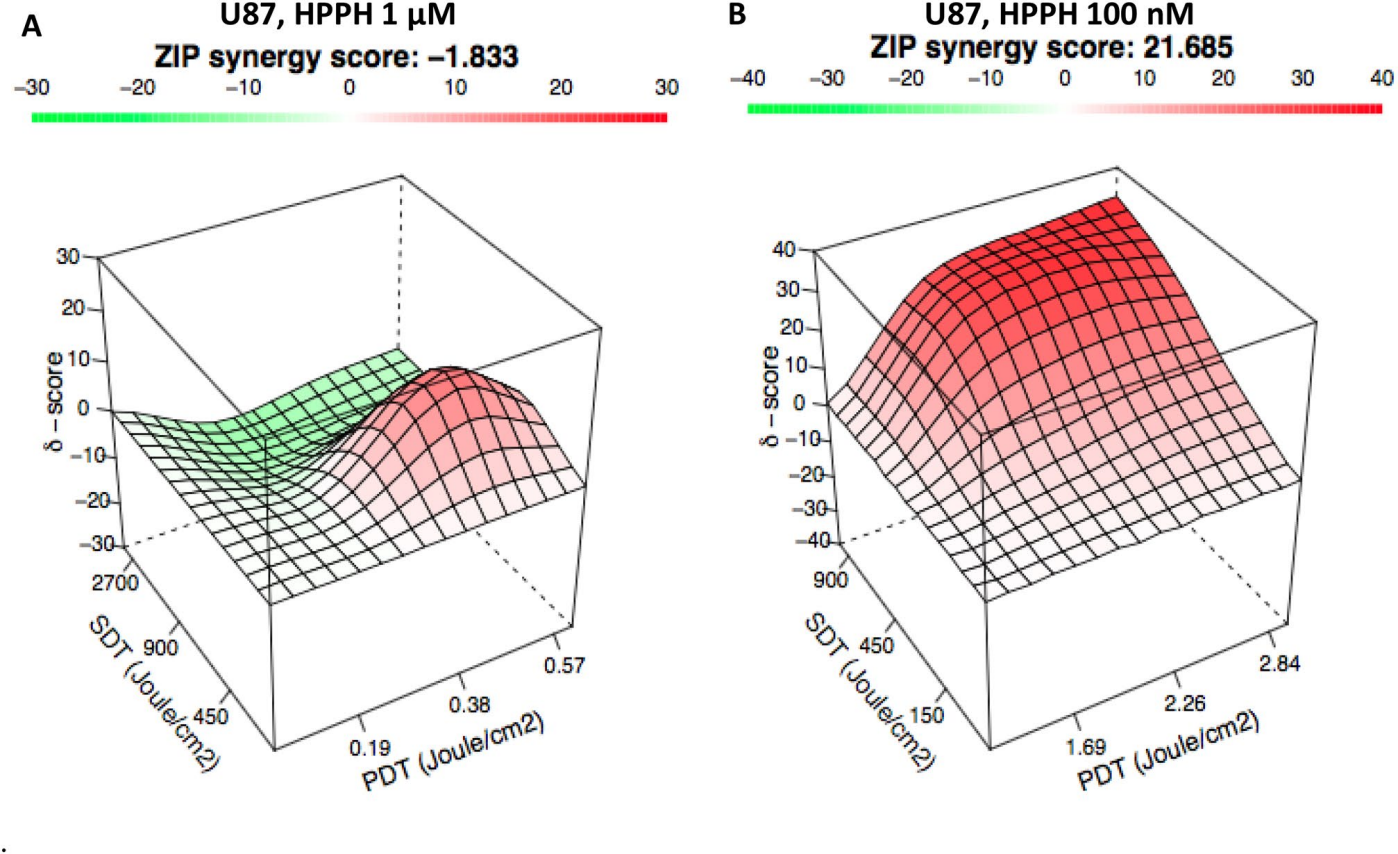
Combination of ultrasound and light yields synergism in vitro response. Cells were plated and incubated with either (**A**) 1 µM or (**B**) 100 nM (**B**) of HPPH. After 24 h of incubation, the cells were exposed to either light, sound, or both. The plates that received both light and ultrasound were first exposed to 665 nm light, then rested for 30 min, then received the dose of ultrasound. 24 h post light/ultrasound exposure, the cells were counted using trypan blue assay. The synergy is calculated utilizing Synergyfinder web application. δ score between − 10 and 10 correspond to an additive response (light green, white and red regions), δ score less than − 10 corresponds to an antagonistic response (dark green), and a δ score greater than 10 corresponds to a synergistic response. No combination of SDT and PDT yields an antagonistic response, but 2700 J/cm of SDT and 0.36 J/cm of PDT yielded the lowest δ score of − 9.49.

After demonstrating that HPPH can be activated via ultrasound, our quest was to determine if there is any interaction of PS with US to induce the cell death. It is well established that PSs generates reactive oxygen species (ROS) via two distinct pathways on exposing with an appropriate wavelength of light, called Type-I or Type-II reactions. In Type-I, the photosensitizer at excited triplet state transfers either an electron or a hydrogen to surrounding molecules to form radical species directly. Whereas, in Type-II reaction, the energy released from the excited triplet state directly converts molecular oxygen to produce singlet oxygen. During photodynamic therapy, HPPH generates ROS primarily through the Type-II reaction^55^ and is highly dependent on oxygen to be effective. To determine if SDT is equally dependent on oxygen and utilizes singlet oxygen to induces cell kill, the ROS generated after exposing the PS by US was measured in vitro using either (a) carboxy-DCFDA, known for detection of radical ions or (b) SOSG (singlet oxygen sensor green) for singlet oxygen. Cells were grown in a 6 well-plate until confluent, then incubated with 5 µM of HPPH. After 24 h, cells were washed and 10 µM of carboxy-DCFDA was added along with serum free media and incubated for 30 min. The cells were then exposed to US for 60 min at 0.5 W/cm^2^, washed and imaged using a fluorescent microscope (Fig. 4A). Cells incubated with PS and exposed to US had significantly increased ROS when compared to the control cells which had no PS and exposed to US. Since carboxy-DCFDA does not effectively detect singlet oxygen, SOSG was added to serum free (SF) media containing 5 µM of PS then exposed to either US or light alone to check for singlet oxygen production. A control experiment was also performed using H_2_O_2_, known for generating hydroxy radical ions. As expected, the light triggered SOSG fluorescence, while cells in presence of H_2_O_2_ had no impact. Interestingly, the mixture that was exposed to US also did not increase in fluorescence (Fig. 4B). These results suggest that SDT follows Type-I ROS generation pathway, and therefore, the presence of oxygen is not required to induce cell death. To test this hypothesis, cells were grown in 35 mm plates and incubated with 1 µM of PS. After 24 h incubation with the PS, some of the plates were placed in a hypoxic chamber at 1% O_2_ and 5% CO_2_ for 2 h to deplete the oxygen^56^. These cells were then exposed to either light or US. At 24 h after exposure, the cells were counted using a trypan blue assay. As expected, the light exposure (PDT) did not induce any decrease in viability due to the lack of oxygen, needed to induce singlet oxygen (Fig. 4D). However, under both normoxic (Fig. 4C) and hypoxic conditions, the US exposure (SDT) was able to induce an equivalent amount of cell death. It is well established that PDT depletes the molecular oxygen in tumors, which does not provide any significant clinical benefits of repeated light exposures. Our study suggests that the limitation of PDT can be resolved by using PDT/SDT combination, and it should produce improved long-term tumor cure by producing singlet oxygen and radical ions after exposing the PS with light and ultrasound (Fig. 4E). To further confirm the additive vs synergetic response of the combination approach, a synergism experiment in a well-established in vitro model was performed (Fig. 5).

For this study, U87 cells were plated as previously descried, however this time some plates were also exposed to light with or without US. After evaluating the cell-viability, the results were tabulated and imputed into the web application Synergy Finder to determine the δ score for each combination^57^. An additive response was observed for most of the combinations of PDT and SDT (δ-score of − 10 to 10 are considered additive, > 10 is synergistic, and < − 10 is antagonistic). While there was no combination that leads to an antagonistic response, high ultrasound exposure and low light exposure had a δ score of − 9.49 that borders antagonism. However, there was some synergistic effect (maxima δ score of 16.15) at high light exposure and low US exposure (Fig. 4A). To further investigate the PS concentration mediated interaction, a second experiment was conducted at lower PS concentration (100 nM). These experiments yielded a high degree of synergy across all doses (Fig. 2B), which could be due to the production of different reactive oxygen species (singlet oxygen and radical ions) responsible for the cytotoxic activity. Porphyrin-based compounds are known to aggregate at higher concentrations due to p–p interaction within the molecules. The phenomenon results in energy and/or electron transfer within the molecules on excitation of PS by light, which results in lower production of reactive oxygen species, and fluorescence. Therefore, in our present study, at a lower concentration of the PS a synergistic impact of PDT and SDT was observed due to reduced aggregation. However, to confirm this hypothesis, a detailed study with a series of effective PS at variable concentrations, light and ultrasound doses is currently underway.

Under in vivo PDT treatment conditions, most of the tetrapyrrole-based PSs tend to circulate in blood for a long time, slowly disaggregate with time, and show optimal uptake in tumors at 24–48 h post-injection before exposing the tumors with either light and/or ultrasound, Therefore, it could be quite possible that compared to in vitro*,* the in vivo PDT/SDT treatment provides improved long-term cure in vivo due to disaggregation of the PS with increased production of ROS.

### In vivo tumor temperature during SDT

**Figure 6:**
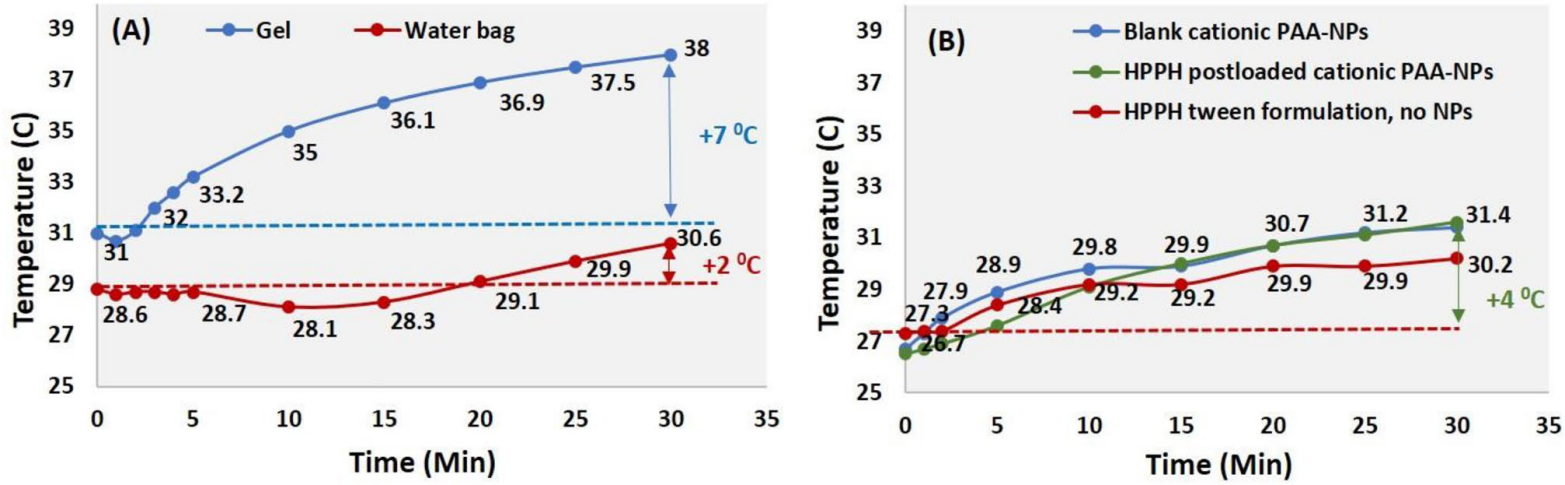
In vivo U87 Glioma tumor temperature during 30 min continuous US exposure (0.5 W/cm^2^, 3.3 MHz) (**A**) Gel applied either on top or tumor or was covered with a water bag [control experiment] and (**B**) either blank cationic PAA-NMe_3_^+^ NPs (10 mg/ml) or HPPH post-loaded cationic PAA-NMe_3_^+^ NPs formulation, or HPPH Tween-80 formulation (HPPH conc: 0.47 µmol/kg) were injected intravenously. After 24 h post-injection, the tumors covered with a water bag were irradiated with US (0.5 W/cm^2^, 3.3 MHz) and the tumor temperature was measured at from 0 to 30 min in 1 min intervals.

Even though in vitro studies demonstrate a synergistic response, the impact of US on a mouse model was investigated before carrying out the in vivo combinational studies of PDT and SDT. It has been observed that high US intensity during treatment increases the temperature of the probe significantly. To determine if the increase in temperature produces any negative impact in tumored mice, the temperature was measured using a probe implanted subcutaneously, and two different mediums (gel and water bag) for the US delivery were used. The use of gel as a medium for US (0.5 W/cm^2^, 3.3 MHz), showed a significant increase of temperature (7 °C). However, when the experiment was performed using a water bag, an increase of less than 2 °C in tumor temperature was observed (Fig. 6A). The increase in temperature can be attributed to the gel used as a medium for the US irradiation, which heats up and causes the heat energy to U87 tumor. This localized hyperthermia can be utilized in future to increase SDT’s efficacy, but in the present study a water bag was used to avoid the complexity caused by temperature in tumor microenvironment. Experiments were carried out to investigate the influence of cationic PAA-NMe_3_^+^ NPs and PS (HPPH) using a water bag as a medium for the US exposure (Fig. 6B). The experimental results indicate that both NPs and PS play a significant role during in vivo SDT. An increase of 3–4 °C was observed with blank cationic PAA-NMe_3_^+^ NPs as well as HPPH post-loaded cationic PAA-NMe_3_^+^ NPs and 3.5 °C with the HPPH Tween-80 formulation. The changes in tumor temperatures during in vivo SDT were monitored by an Omega microprocessor thermometer (*Model HH23, Type J-K-T Thermocouple*).

We have previously shown^58^ that whole body or local hyperthermia enhances the PDT efficacy of HPPH due to increased uptake of PS in tumor. A variety of gold NPs have also been used for local hyperthermia for cancer therapy. This approach requires high dose of radiation, which is known to damage the normal tissues also^59^. The in vivo SDT conditions discussed herein, water was used as a medium, which yielded limited increase in tumor temperature, and thus the hyperthermia should not have any role in providing enhanced long-term tumor cure.

### In vivo blood–brain barrier (BBB) permeability of HPPH-NPs

**Figure 7:**
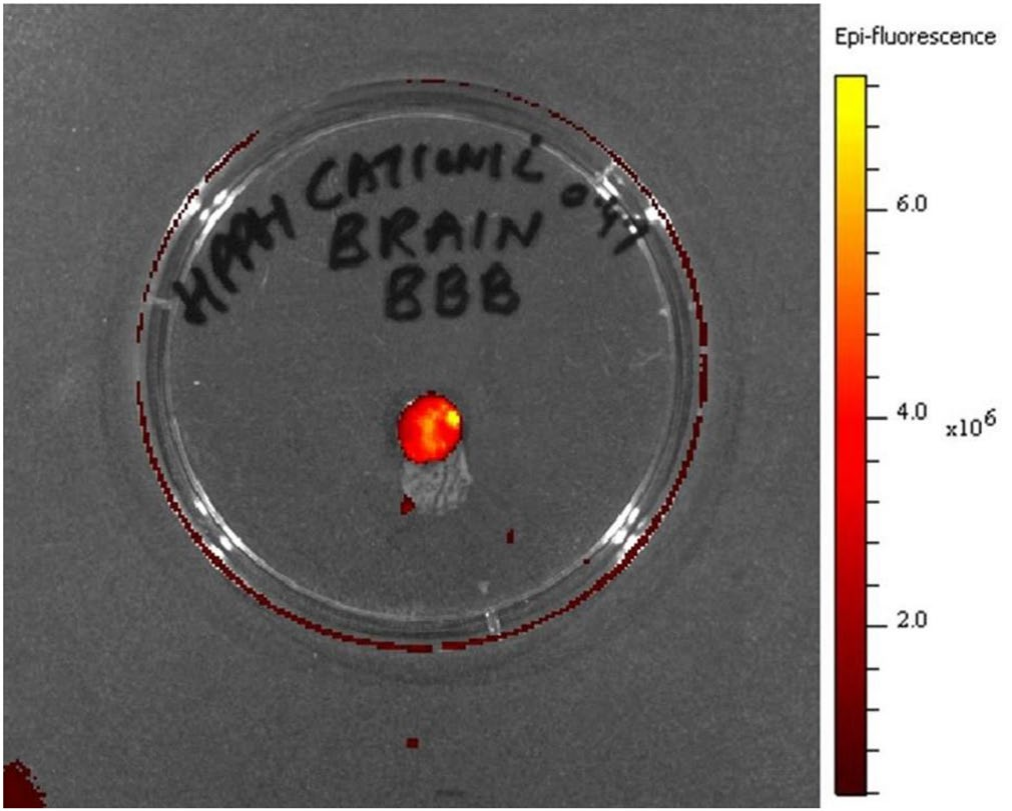
HPPH in cationic PAA-NMe_3_^+^ NPs (dose: 0.47 μmol/kg was intravenously injected in a normal BALB/c mouse. At 24 h post-injection, the brain of the mouse was removed, placed in a petri dish and imaged by IVIS Spectrum [Ex (HPPH): 675 nm, and Em: 720 nm], indicating that HPPH in nano-formulation crosses the intact blood brain barrier (BBB).

One major hurdle in treatment of GBM is the blood–brain barrier (BBB). The BBB is comprised of endothelial cells layer with tight junctions that prevents molecules from entering the brain. The BBB prevents many drugs from accumulating in the brain at therapeutic levels, thus lowering the efficacy of standard chemotherapy on malignant brain tumors^60,61^. The BBB permeability of HPPH post-loaded cationic PAA-NMe_3_^+^ NPs was investigated in vivo using BALB/c mice. The mice were administrated with the HPPH post loaded cationic PAA-NMe_3_^+^ NP formulation at a therapeutic dose of 0.47 μmol/kg a tail vein injection. After 24hrs, the brain tissues of the mice were harvested and imaged with an in vivo optical imaging system (PerkinElmer IVIS Spectrum). As shown in Fig. 7, fluorescence signal of HPPH was observed at 24 h (wavelength: Ex675/Em720). This study demonstrates that HPPH loaded NPs penetrate the BBB and can be used for both PDT and combinational PDT-SDT treatment of brain cancer.

### In vivo tumor uptake vs. PDT and PDT-SDT efficacy of the PS and PS-NPs

**Figure 8:**
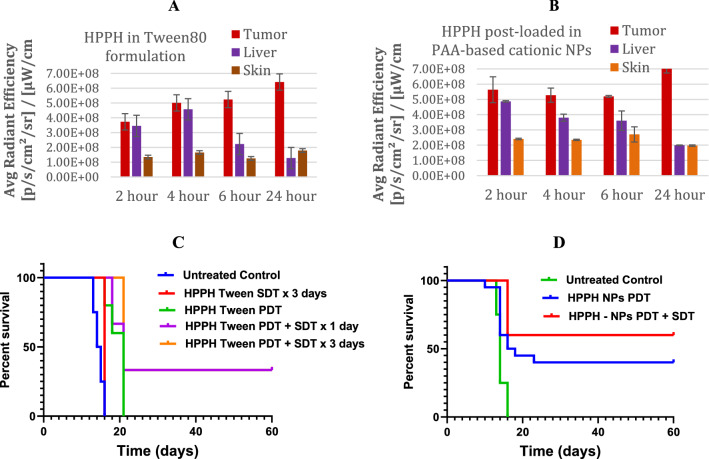
Comparative tumor uptake of HPPH in (**A**) Tween80 formulation, and (**B**) HPPH post-loaded in PAA cationic NPs at 2, 4, 6 and 24 h post-injection (HPPH in Tween or PNPs formulation at a dose of 0.47 μmol/kg). Antitumor activity of PDT and PDT + SDT combination of HPPH (0.47 μmol/kg) formulated either (**C**) in Tween80 or (**D**) post-loaded in PAA cationic NPs in SCID mice bearing U87 glioma xenografts: Compared to HPPH Tween-80 formulations at a dose of 0.47 μmol/kg, the HPPH post-loaded in cationic PAA-NMe_3_^+^ at the same dose showed significantly enhanced long-term tumor cure with no mortality. Light dose: 665 nm, 135 J/cm^2^, 75 MW/cm^2^) at 24 h post‐injection of PS), US dose: 0.5 W/cm^2^, 3.3 MHz, 30 min. HPPH-PAA NPs in combination of PDT & SDT gave 60% tumor cure (6/10 SCID mice were tumor free on day 60, *p*-value 0.04.

For this study, SCID Prkdc mice were tumored with U87 glioma xenografts in the flank to determine the HPPH uptake, and efficacy of PDT, SDT, and PDT + SDT using HPPH formulated in both tween and NPs. Once the tumors were of treatment size, mice were injected with either formulation at 0.47 µmol/kg. The tumor uptake of HPPH, Tween80 and NPs formulation is shown in Fig. 8A,B indicate that NPs formulation showed increased tumor-uptake of HPPH. However, both formulations gave highest uptake at 24 h post-injection (Fig. 8C,D). After whole body fluorescence imaging, the tumors were exposed to light (PDT) or light and ultrasound (SDT). The HPPH in Tween formulation shows that PDT + SDT had complete response (CR) 36% on day 60. Increasing the number of SDT treatments had no impact in cure rate (CR). When similar experiments were carried out using cationic PAA-NMe_3_^+^ NP formulations, enhanced PDT efficacy as well as improved combinational effect was observed. From the results summarized in Fig. 8D, the PDT + SDT achieved a complete response of 60.0% compared to the CR of 36% observed with PDT alone. It is evident that when HPPH on subjecting to a combination therapy of PDT followed by SDT, intense tumor-necrosis was observed with notable improvement on long-term tumor cure. The statistical evaluation was performed using Mantel-Cox test and the *p*-value: 0.0003 (≤ 0.04) of the survival curves between HPPH formulated in Tween-PDT alone and the p-value: 0.04 (≤ 0.05) between HPPH post-loaded in cationic PAA-NMe_3_^+^-PDT alone *vs*. PDT & SDT combination was statistically significant.

### Impact of PDT/SDT in tumor vasculature

**Figure 9:**
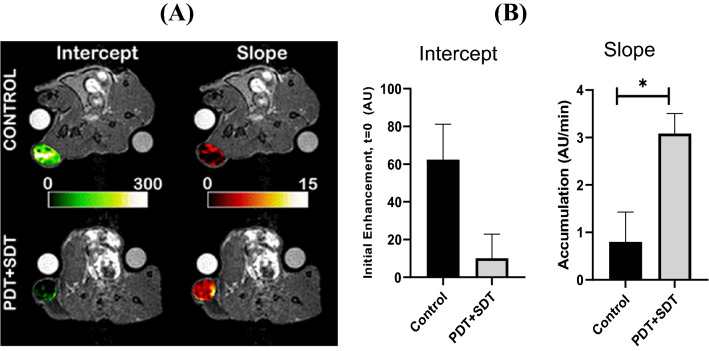
Assessment of tumor vascular changes by MR imaging. (**A**) Pseudo-colorized dynamic enhancement maps of a control mouse (top) and treated mouse (bottom) calculated on a voxel-by-voxel basis. A clear reduction in the blood flow in the tumor (intercept) and increased permeability (slope) in the treated mouse. (**B**) Grouped analysis between control and treated animals (n = 3 each). A substantial reduction in initial enhancement (intercept) was observed following PDT + SDT treatment, as well as increased permeability (slope) (*p = 0.039, two-tailed Student’s t-test).

The combination of PDT followed by SDT appears to strongly affect tumor vasculature, relative to the control group, as determined by dynamic contrast-enhanced imaging using the macromolecular agent HSA-Gd(III)DTPA^62^. Regression analysis revealed large differences in the slope of the enhancement curve (permeability) and intercepts (vascularity) between the treated group and controls as shown in Fig. 9. A 76% reduction in the initial enhancement was observed for the treated group (p = 0.083, two-tailed Student’s t-test), while accumulation rate of the contrast in the treated group was 286% higher than the control group (p = 0.039). These changes indicate acute losses in the tumor vascular integrity as a result of PDT + SDT therapy, exemplified through a combination of increased permeability and vascular shutdown leading to tumor destruction.

### Cationic PAA NPs did not show toxicity in BALB/c mice

**Figure 10:**
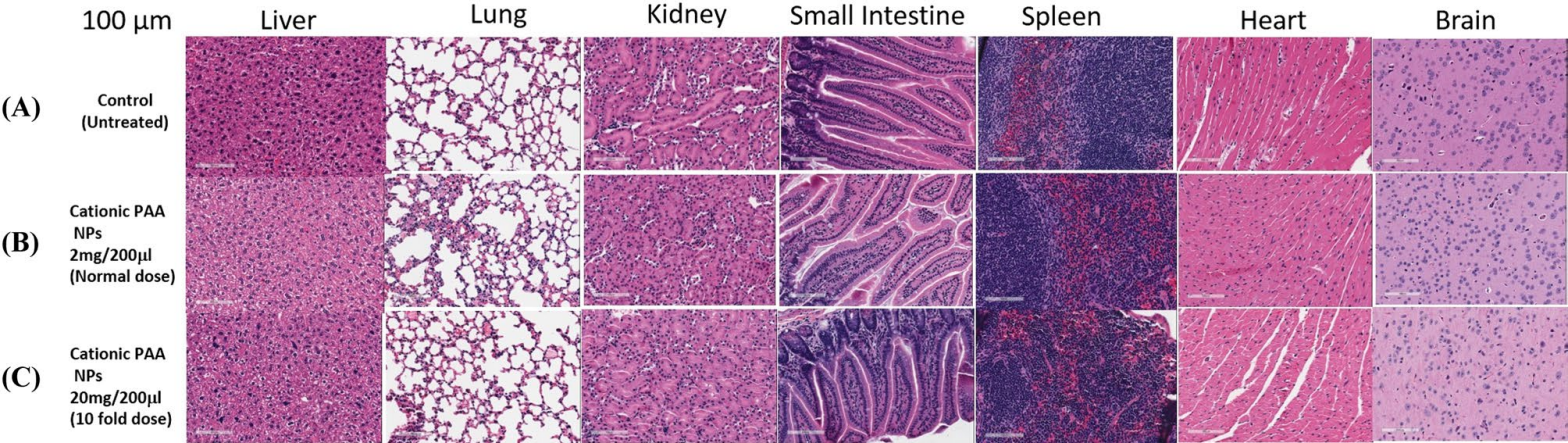
Representative histopathological staining of various organs (100 μm thickness) of BALB/c mice (**A**) without injecting the PAA cationic NPs to mice (control) and at day 14 post-injection of the NPs at a dose of (**B**) 2 mg/200 μλ/mouse and (**C**) 20 mg/200 μλ/mouse (tenfold higher dose of the NPs requires to inject (i. v.) a desired therapeutic dose (0.47 μmole/kg) of HPPH. No organ toxicity was observed even at tenfold higher dose of the NPs used for injecting a therapeutic dose (0.47 μmole/kg) of HPPH. The weights of mice used in this study were in the range of 20–24 g/mouse.

The comparative histological images of each organ with the control group and at a dose of 2 mg in 200 μl, 20 mg in 200 μλ, which is tenfold higher than the therapeutic dose of the NPs used in HPPH formulation are shown in Fig. 10. The scale bar in the image represents 100 μm. The organs did not show any pathological changes between the normal/untreated controls and the samples from the mice treated at these doses. No significant changes were observed in the histology analysis of normal and NP treated organs. Also, there was no cellular degeneration or displacement on the cellular level. The comparison illustrates no organ toxicity even at tenfold higher dose of the NPs used for injecting a therapeutic dose (0.47 μmole/kg of HPPH) was observed by Cationic PAA nanoparticles in this study.

## Methods

### Selection of ultrasound dose

The doses and schedules: single US dose of 0.5 W/cm^2^, 3.3 MHz at variable time points.

### In vitro PDT-SDT Efficacy of HPPH

U87 cells were grown to confluency on 5 cm plates, and then incubated with 1 µM or 100 nM of the HPPH. After 24 h, the plates were exposed to 0.5 W/cm^2^ ultrasound (US) for a total of 60 or 90 min. The viability of each group was determined by harvesting the cells utilizing trypsin EDTA and counting the cells using trypan blue staining 24 h after exposure^63^. For synergy experiments, cells were exposed to 5, 15, 30, 60 or 90 min of ultrasound at 0.5 W/cm^2^ and 665 nm light at 565 mW over a 15 cm spot at a dose rate of 3.2 mW/s for 5, 10, 15, 45, 60, 75 s. J/cm^2^ was calculated using the formula: mW/cm^2^/1000 × time (s).

### In vitro ROS determination with carboxy-DCFDA

Cells were plated in a six well plate and allowed to grow to confluency before adding 5 µM of HPPH. After 24 h incubation, the cells were washed twice with cold PBS and serum free media was replaced, loaded with 10 µM of carboxy-DCFDA. After 30 min incubation, the wells were either exposed to US (30 min at 0.5 W/cm^2^) or H_2_O_2_ (positive control). After 30 min, the wells were washed again with PBS and Hoechst 33342 was added before imaging with a Zeiss fluorescent microscope. The fluorescence observed at Excitation: 410 nm/Emission 675 nm was measured using ImageJ and divided by the number of cells in the picture to determine Normalized Grey Value.

### Determination of in vivo PDT and SDT efficacy

SCID^prkde^ mice with subcutaneous U87 xenografts of 200–250 mm^3^ were injected intravenously (i.v) with HPPH at dose of 0.47 μmol/kg in either Tween or NP formulations (the HPPH dose was selected on our previous in vivo studies of HPPH-PDT in other cancer types). The PS uptake in U87 tumors was determined by fluorescence imaging using a PerkinElmer IVIS Spectrum at variable timepoints, and the maximum uptake was observed at 24 h post-injection. Therefore, at this timepoint the tumors were irradiated with light (fluence: 135 J/cm^2^; fluence rate: 75 mW/cm^2^) for 30 min at 665 nm using a Lightwave laser diode. Mice were restrained without anesthesia in plexiglass holders designed to expose only the tumor and a 2–4 mm annular margin of skin to light. For SDT exposure, continuous US dose of power 0.5 W/cm^2^ and 3.3 MHz was administered as described above. For the first 10 days, tumor measurements were taken daily, then three times a week for 4 weeks, and finally twice a week thereafter for a total of 60 days post treatment. Two axes (mm) of tumor (L, longest axis; W, shortest axis) were measured with the aid of a vernier caliper. Tumor volume (mm^3^) was estimated using a formula: tumor volume = ½ (L × W^2^). The complete tumor regression (CR) was defined as the inability to detect tumor by palpation at the initial site of tumor appearance for more than 2-month post-therapy. The partial tumor regression (PR) was defined as ≥ 50% reduction in initial tumor size. Observations of edema, erythema, and scar formation in the treatment field was observed and recorded. Tumor response for each treatment was compared to tumor-bearing animals not subjected to therapy (PDT, SDT, or PDT + SDT). The optimization of PS, light and US doses is currently underway.

### Toxicity of cationic PAA NPs

Cationic PAA NPs in PBS formulation were injected intravenously to normal BALB/c mice (3 mice/group) at three different doses (2 mg in 200 μλ, 10 mg in 200 μλ and 20 mg in 200 μλ). The mice were kept at room temperature with proper maintenance following the guidelines of IACUC approved protocol for 14 days and were checked every day for physical appearance and weight loss, and other physical abnormalities. On day 14 tissue samples of liver, lung, kidney, small intestine, spleen, heart and brain were collected, and submitted for histopathology analysis with a control group of normal mice without injecting any NPs. For determining the organ toxicity of NPs, routine hematoxylin and eosin staining were used on formalin-fixed, paraffin-embedded tissues. Histopathological changes of tissues after the treatment of cationic PAA NPs were analyzed and compared to untreated controls to determine drug-induced toxicity.

### MR imaging

The effects of treatment on tumor vasculature were examined using dynamic contrast-enhanced MR imaging at 4.7 T (35 mm ID coil, Bruker Biospin, Billerica MA). Two 5 mm NMR tubes containing 1% agarose with CuSO4 concentrations of 1 mM and 2 mM were included for signal normalization and a macromolecular contrast agent, Gd(III)DTPA covalently bound to human serum albumin, HSA-Gd(III)DTPA, was used for dynamic enhancement. Baseline scans were acquired using a 3D, spoiled gradient recalled echo scan (SPGR, TE/TR/FA = 3/15/40, FOV = 48 × 32 × 32 mm, matrix = 192 × 96 × 96, NEX = 1). Following baseline acquisitions, HSA-Gd (III)DTPA was injected via tail vein at a dose of 50 μmol [Gd]/kg, and seven additional SPGR scans were acquired post-injection to characterize the initial enhancement and accumulation rate over approximately 20 min. Due to its large molecular weight, HSA-Gd (III)DTPA is retained in normal vasculature but will extravasate from immature or permeable vessels at a near-linear rate^55^.

MR images were reconstructed to an isotropic voxel size of 187 microns and normalized using agarose phantom signal intensities. Intratumoral signal intensities were sampled from regions of interest using commercially available software (Analyze 10.0, AnalyzeDirect, Overland Park KS) and increases in normalized tumor signal intensities were calculated by subtracting baseline values from post-injection data. Linear regression was applied to the changes in signal vs time post-injection to calculate the rates of accumulation (slope) and initial enhancement (intercept), extracting vascular permeability and fractional vascular volumes, respectively. To visualize these parameters, 3D datasets were first filtered with a 3 × 3 × 3 low pass filter and appended into a 4D dataset, and then linear regression was applied per voxel across the 4th dimension.

### Statistical analysis

The standard log-rank test (Mantel-Cox) was used for statistical analysis. It is a hypothesis test and compares the survival based on Kaplan Meier survival curve. It is a test of significance to detect difference between groups to confirm if one group has risk of an event greater than the other. For analyzing the in vivo PDT/SDT efficacy (cure), the survival curves were plotted using the drug dose over tumor regrowth.

### Ethical approval for using animals

The in vivo experiments discussed in this manuscript were performed in compliance with all state, local, federal laws and the PHS Policy on the Human Care and use of Laboratory Animals. This study was conducted in an AAALAC accredited facility. The animal study was approved by the corresponding ethics committee in the manuscript.

## Conclusions

We have demonstrated that HPPH is an effective photo and sonosensitizer under in vitro and in vivo conditions. Additionally, due to SDT relying on Type-I generation of ROS, which is oxygen independent, PDT and SDT combinations yield synergistic effects. These in vitro results also supported the in vivo outcome, where the HPPH PDT + SDT therapy produced enhanced tumor cure. Interestingly, the HPPH post-loaded in PAA-NMe_3_^+^ NPs were quite promising due to multiple reasons. Primarily, the use of light or US triggers the local release of PS and should reduce the presence of the PS in other organs. Additionally, the NP formulation increases tumor cell-specificity, uptake and efficacy in both in vitro (viability of 57% vs 43% comparing Tween formulation to NP) an in vivo (0% CR using Tween formulation vs 36% CR using NP). The nontoxic NPs (toxicity of various organs in BALB/c mice was confirmed by histopathology analysis) also able to cross the blood–brain–barrier (BBB), thereby demonstrating that the formulation will be an effective vehicle for further in vivo experiments using orthotopic models. The synergy may also occur as PDT & SDT has shown to increase capillary permeability as demonstrated by MR imaging. The combined PDT and SDT treatments were able to significantly increase CR rate of U87 tumors in SCID mice model from 36% using PDT alone to 60% post 60 days of therapy, and these results are quite promising. However, further studies are required to optimize the drug, light and US doses, and to determine the impact of slight increase in tumor temperature in long-term tumor response, before moving this approach from bench to bedside for the treatment of glioblastoma and other cancer types, which are currently underway.

## Supplementary information

Supplementary Information.

## Abbreviations

GBM: Glioblastoma multiforme
ALA: Aminolaevulinic acid
PP IX: Protoporphyrin-IX
ROS: Reactive oxygen species
US: Ultra-sound
SS: Sonosensitizers
SDT: Sonodynamic therapy
PS: Photosensitizers
PDT: Photodynamic therapy
NIR: Near-infrared
NP: Nano-particle
HSA: Human serum albumin
PBS: Phosphate-buffered saline
AF-PAA: Amine-functionalized polyacrylamide nanoparticles
SOSG: Single oxygen sensor green
DCFDA: Dichlorodihydrofluorescein diacetate
TMZ: Temezolomide
VEGF: Vascular endothelial growth factor
MRI: Magnetic resonance imaging
FDA: Food and Drug Administration
