# Supplementary material for: Sonodynamic therapy in combination with photodynamic therapy shows enhanced long-term cure of brain tumor
Source: Sci Rep. 2020 Dec 11;10:21791. doi: 10.1038/s41598-020-78153-0 (PMC7732989; doi:10.1038/s41598-020-78153-0)
Supplement: Supplementary file 1 — Supplementary Information. [file 41598_2020_78153_MOESM1_ESM.pdf]

Supporting Material Information

Sonodynamic Therapy in Combination with Photodynamic Therapy Shows Enhanced Long-term Cure of Brain Tumor

*Ballav M. Borah, Joseph Cacaccio, Farukh Durrani, Wiam Bshara, Steven Turowski, Joseph Spernyak and Ravindra K. Pandey**

**E-mail addresses: ravindra.pandey@roswellpark.org*

List of Contents:

1. Synthesis of amine functionalized (AF) cationic polyacrylamide (PAA) NPs Page S2
2. Table S1 – List of reagents for the preparation of PAA NPs Page S3
3. Figure S1: Absorption spectra of HPPH with and without nanoparticles Page S4
4. Figure S2. In vitro cell viability of HPPH-Tween and HPPH-PAA NPs formulations Page S5
5. Stability of PS post-loaded to cationic PAA NPs Page S6-S8
    - Figure S3. Size of cationic PAA NPs
    - Post-loading of PS to AF-PAA NPs
    - Formulation of PS in Tween 80
    - Quantification of Loading Efficiency (Figure S4)
6. Photophysical characterization of HPPH-post-loaded cationic NPS Page S8- S9
7. Table S2: US-triggered rate of release of PS (HPPH) from PS-post-loaded NPs Page S10

Synthesis of amine functionalized cationic polyacrylamide (PAA-NMe_3^+) nanoparticles:

Anhydrous hexane (45 mL) was stirred under argon for 1 hour at room temperature to de-gas the liquid. Dioctyl sulfosuccinate sodium salt (1.6 g; 3.6 mmol) was added and the argon atmosphere was restored. 3.3 ml of Brij-30 was injected with a syringe and the mixture was stirred for 30 min under constant purge of argon. To this mixture, a solution of acrylamide (711 mg; 10.0 mmol), [3-(Methacryloylamino) propyl]trimethyl-ammonium chloride (129.15 μl; 0.50 mmol) in 1.3 ml 18 MΩ argon-purged water, and biodegradable 3-acryloxy-2-hydroxypropyl methacrylate (375 µl; 428 mg; 2.0 mmol) was added. The solution was injected into the reaction flask with a syringe and mixed for 30 min under constant purge of argon. Polymerization was initiated by adding freshly prepared ammonium persulfate aqueous solution (10% w/v aqueous solution, 150 µL) and TEMED (150 µL) (**Table S1**). The resulting solution was stirred vigorously overnight. At the completion of polymerization, hexane was removed by rotary evaporation and the particles were precipitated by addition of ethanol (50 ml). The surfactant and residual monomers were washed away from the particles with ethanol (150 ml), followed by washing with water (100 ml) five times each in an Amicon ultra-filtration cell equipped with a Biomax 300 kDa cutoff membrane. The concentrated NPs were lyophilized prior to use.

Materials:

Acrylamide, N,N,N′,N′-tetraethylmethylenediamine, ammonium persulfate, polyethylene glycol dodecyl ether (Brij L4), 3-(acryloyloxy)-2-hydroxypropyl methacrylate, hexane, and dioctyl sulfosuccinate (AOT) were purchased from Sigma-Aldrich (St. Louis, Missouri). The 3-(aminopropyl) methacrylamide was obtained from Polysciences Inc. (Warrington, Pennsylvania), and ethanol (200 proof) was obtained from Fisher Scientific (Pittsburgh, Pennsylvania). Nanosep 100 K Omega filters were purchased from Pall, Port Washington, New York. Amicon ultra-filtration cell equipped with a Biomax 300 kDa cutoff membrane was purchased from Millipore, Billerica, Massachusetts.

Table S1. List of reagents for the preparation of AF-PAA nanoparticles

Reagent	Amount	Purpose
Hexane, anhydrous 95% CAS: 110-54-3	45ml	Solvent
Dioctyl Sulfosuccinate (AOT) CAS: 577-11-7	1.6g	Anionic surfactant. Disbursing agent. Used to prepare reverse micelles
Polyoxyethylene lauryl ether (Brij L4) CAS: 9002-92-0	3.1g or 3.3ml	Nonionic surfactant. Anti-foaming
Acrylamide >99% CAS: 79-06-1	711mg	Attaches in a chain, end-to-end, via the radical reaction
[3-(Methacryloylamino) propyl]trimethyl-ammonium chloride CAS: 51410-72-1	Soln is 50% by wt in H_2O. 68 mg, 129.15 μL	Provides amine functionality (-NMe_3^+ group) but decreases solubility.
3-acryloyloxy-2-hydroxypropyl methacrylate (AHM) CAS: 1709-71-3	428mg or 375μl	Increases solubility, but decreases amino groups; it is the biodegradable cross-linker
Ammonium persulfate (APS) CAS: 7727-54-0	250μl of 10% w/v in water	Creates radicals that initiate the reaction
N,N,N',N'-tetramethylethylene diamine (TEMED) CAS: 110-18-9	250μl	Acting as a bidentate ligand, it stabilizes the radical
18.2MΩ water	1L	Used for washes x 5
95% Ethanol	1L	Used for washes x 5

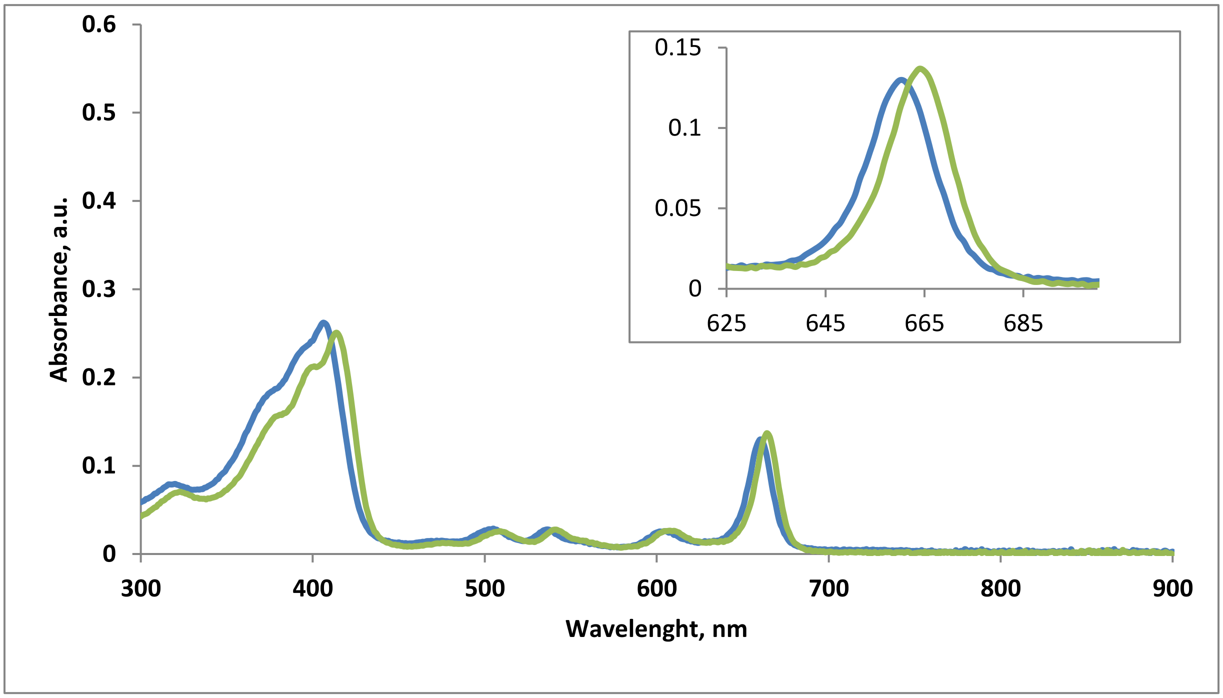

Figure S1: Absorption spectra of HPPH (PS) in methanol (blue line, 660 nm), and post-loaded in PAA-based NPs (green line, 664 nm). HPPH concentration: 2.9 μMol.

<u>Note</u>: The long wavelength absorption of HPPH in the presence of HSA exhibits 5 nm red shift (660 to 665 nm), whereas no change in long wavelength absorption of HPPH in PAA formulation (664 nm) was observed.

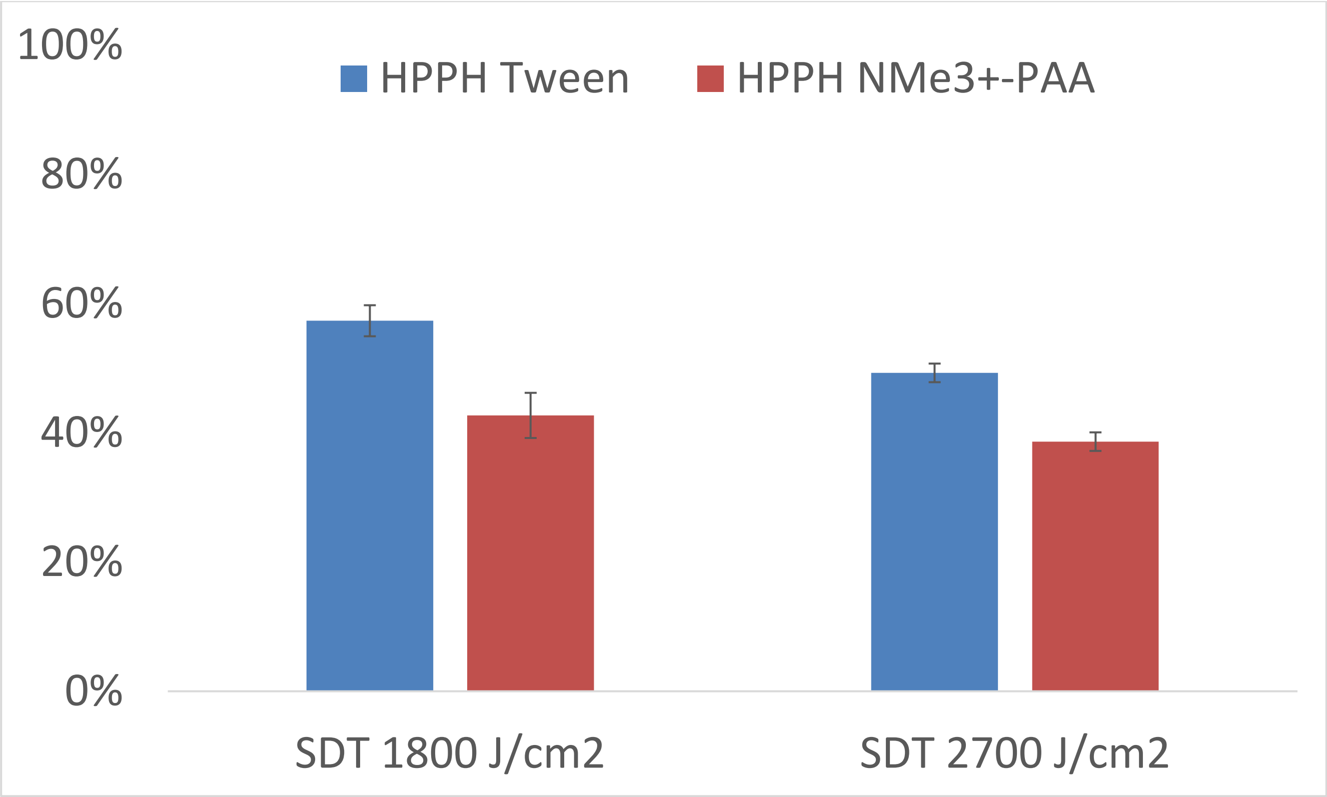

HPPH Tween	Cell Viability
SDT 1800 J/cm^2	57.3%±2.4%
SDT 2700 J/cm^2	47.7%±3.0%

HPPH NMe3+ PAA	Cell Viability
SDT 1800 J/cm^2	42.7%±3.5%
SDT2700 J/cm^2	38.6%±1.4%

Figure S2. *In vitro* Cell Viability, HPPH Tween VS HPPH cationic PAA-NMe_3^+ NP formulations. Viability was determined by harvesting the cells using Trypsin EDTA and counting the cells using trypan blue. Viability is presented as % of control. SDT paramaters are 60 vs 90 min exposre to 0.5W/cm^2 3.3 MHz.

Stability of PS post-loaded to cationic PAA-NMe_3^+ NPs:

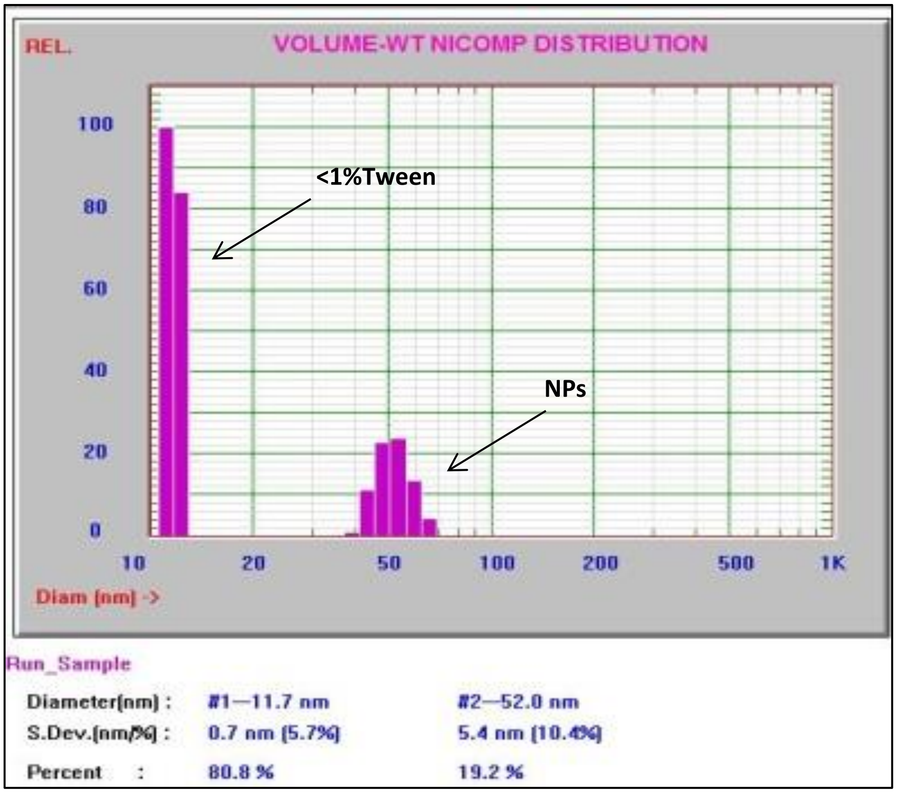

Figure S3. Size of cationic PAA-NMe_3^+ NPs determined by DLS.

A simple, reproducible, highly efficient and a novel approach have been developed for the long-term stability, release and delivery of cancer therapeutics, using lyophilized cationic PAA-NMe_3^+ nanoparticle formulation. (Figure S3). Even after a year, dynamic light scattering (DLS) measurements of the cationic NPs show no increase in size due to aggregation, proving their long-term stability (NPs dispersed in April 2018, stores at 4°C and DLS measured in April 2019).

Materials:

Acrylamide, N,N,N′,N′-tetraethylmethylenediamine, ammonium persulfate, polyethylene glycol dodecyl ether (Brij L4), 3-(acryloyloxy)-2-hydroxypropyl methacrylate, hexane, and dioctyl sulfosuccinate (AOT) were purchased from Sigma-Aldrich (St. Louis, Missouri). The 3-(aminopropyl) methacrylamide was obtained from Polysciences Inc. (Warrington, Pennsylvania), and ethanol (200

proof) was obtained from Fisher Scientific (Pittsburgh, Pennsylvania). Nanosep 100 K Omega filters were purchased from Pall, Port Washington, New York. Amicon ultra-filtration cell equipped with a Biomax 300 kDa cutoff membrane was purchased from Millipore, Billerica, Massachusetts.

Post-loading of Photosensitizers in amino functionalized cationic PAA-NMe$_3^+$ Nanoparticles

The lyophilized cationic PAA-NMe$_3^+$ nanoparticles were dispersed in 1% Tween-80 PBS (pH 7.4, 10 mM) to a final concentration of 10 mg/mL. PSs or chemo agents were dissolved in DMSO to a final concentration of 20 mM, 20 µL of this mixture were added to 2 mL of nanoparticle solution and was stirred for 2 hours. The nanoparticle dispersion were transferred to Amicon Ultra-4 100 kDa centrifuge filter and centrifuged at 4,000 rpm for 80 minutes to remove excess DMSO, Tween-80, and the PS that did not post-load. The absorbance of the filtrate was measured, and if signal for the photosensitizer was detected, the retentate was reconstituted to the original volume with fresh PBS and re-centrifuged.

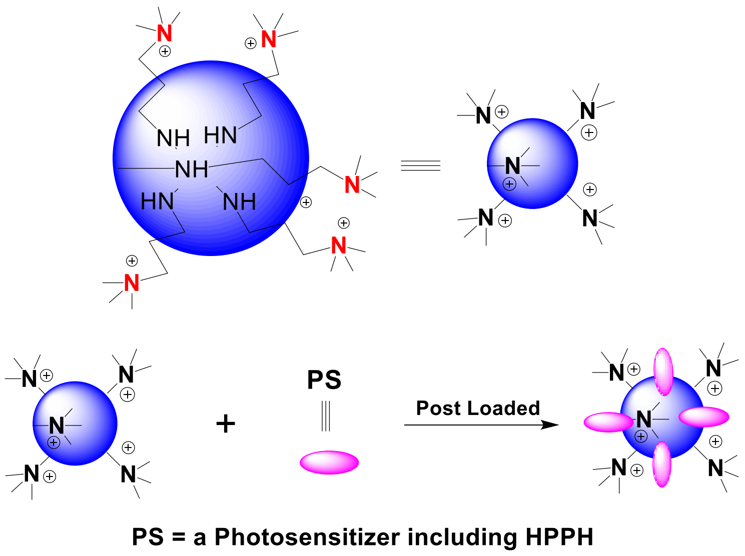

Scheme S1: Post-loading of Ps or Chemotherapeutics in PAA based NPs.

This process was continued until no signal was detectable in the filtrate, spectrophotometrically. The nanoparticle solutions were syringe-filtered through a 0.2 micrometer and stored at 4°C. Concentration could not be measured with nanoparticles in ethanol suspension, as the scatter skewed the absorbance measurement. The concentration was measured by mixing an aliquot of suspension in ethanol, and

centrifugation in a benchtop centrifuge at 14,000 RPM for 1 minute to pellet and remove emptied nanoparticles.

To calculate the concentration of PS within NPs, the PS post-loaded NP is diluted in ethanol and measured spectrophotometrically using a Varian (Cary-50 Bio) with a molar extinction coefficient (ε) of 47,500$L/(mol * cm)$. To remove the scattering in the absorbance spectra, the NPs were centrifuged filtered with a Microfuge membrane-filter (NANOSEP 100K OMEGA, Pall Corporation) at 5,000 rpm for 10 minutes. The NPs are retained above, and the PS loaded within the NP is in the filtrate. The concentration of the PS in filtrate and in NP was measured spectrophotometrically.

Formulation of PS in Tween80:

Drugs were formulated in 1% Tween-80 in PBS. Amount of compound and volume of solution were calculated to generate a 500 µM stock concentration. Drug and necessary volume of Tween-80 were added to a mortar and mulled to a paste. The paste was allowed to sit overnight, mulled again, and then the calculated amount of PBS was added to the paste and mixed. Solution was filtered through a 0.2µm syringe filter and concentration was measured spectrophotometrically. Drug solutions were stored at 4°C when not in use.

Quantification of Loading Efficiency

A suitable amount of dye loaded nanoparticles was suspended in 1% Tween 80 in water solution to measure the UV–vis absorbance (Varian Cary-50 Bio UV-Visible spectrophotometer, Palo Alto, CA). The Beer–Lambert law was used to calculate the concentration of the photosensitizer loaded in the nanoparticles. Fluorescence spectroscopy (FluoroMax-3, Jobin Yvon/SPEX Division, Instruments S. A. Inc., Edison, NJ) was also conducted to verify photosensitizer integrity after entrapment.

Photophysical characterization of HPPH-post=loaded cationic NPs

UV-Vis absorption spectra of compounds (MeOH solutions) were acquired using a Varian (Cary-50 Bio) spectrophotometer. The extinction coefficients of all compounds were determined by weighing a particular amount of solid and dissolving in a 50 ml volumetric flask using methanol as the solvent. First, the molar concentration (*C*) of each solution was calculated from its weight and volume. The

absorbance (A) obtained from the UV-Vis spectrophotometric measurements was used to determine the extinction coefficient (ε) from Beer-Lambert's Law (A = ε*dC*), where *d* is optical path length (cuvette thickness, *d* = 1cm). The presence of HPPH post loaded in cationic PAA-NMe_3^+ nanoparticle was observed using UV-Vis absorbance spectroscopy. Suspensions containing the respective nanoparticles (10 mg/ml) were prepared in 1% Tween-80 in 18 MΩ H2O and filtered through a 0.8 micron filter. The UV-Vis spectra were collected from 1000 nm to 300 nm using an 18 MΩ water blank in quartz cuvettes. The absorption of resulting supernatants from the 18 MΩ H2O and ethanol washes, during preparation of the modified nanoparticles, was obtained. Using the extinction coefficient of HPPH (□: 47,500 $Lmol^{-1}cm^{-1}$ at 665 nm), the concentrations of unreacted HPPH and then the molar amounts were calculated

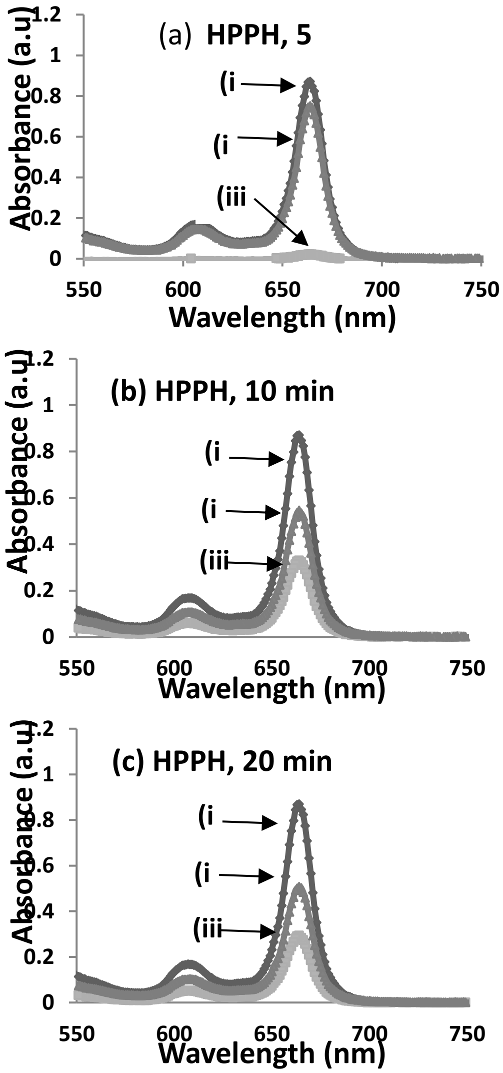

Figure S4. UV-Vis spectra of HPPH (a-c) post-loaded Cationic PAA NPs after 5, 10 and 20 mins of US irradiation respectively. (i) original, (ii) retentate and (iii) filtrate.

Table S2: US triggered release rate of PS (HPPH) from its post-loaded cationic PAA-NMe_3^+ NPs formulation

	**% Released**	**Standard Error (Filtrate)**
**5 Min**	2.4	0.06
**10 Min**	38.7	0.38
**20 Min**	70.8	0.21

0.5 W/cm2 power, 3.3 MHz
